# On predicting regulatory genes by analysis of functional networks in C. elegans

**DOI:** 10.1186/s13040-015-0066-0

**Published:** 2015-11-02

**Authors:** Olga V. Valba, Sergei K. Nechaev, Mark G. Sterken, L. Basten Snoek, Jan E. Kammenga, Olga O. Vasieva

**Affiliations:** 1 LPTMS, Université Paris Sud, Orsay Cedex, France; 2 National Research University, Higher School of Economics, Moscow, Russia; 3 P.N. Lebedev Physical Institute of the Russian Academy of Sciences, Moscow, Russia; 4 Institute of Integrative Biology, University of Liverpool, Liverpool, UK; 5 Laboratory of Nematology, Wageningen University, Wageninge, Netherlands

**Keywords:** Networks, eQTL, The shortest paths, Regulatory genes, Longevity

## Abstract

**Background:**

Connectivity networks, which reflect multiple interactions between genes and proteins, possess not only a descriptive but also a predictive value, as new connections can be extrapolated and tested by means of computational analysis. Integration of different types of connectivity data (such as co-expression and genetic interactions) in one network has proven to benefit ‘guilt by association’ analysis. However predictive values of connectives of different types, that had their specific functional meaning and topological characteristics were not obvious, and have been addressed in this analysis.

**Methods:**

eQTL data for 3 experimental C.elegans age groups were retrieved from WormQTL. WormNet has been used to obtain pair-wise gene interactions. The Shortest Path Function (SPF) has been adopted for statistical validation of the co-expressed gene clusters and for computational prediction of their potential gene expression regulators from a network context. A new SPF-based algorithm has been applied to genetic interactions sub-networks adjacent to the clusters of co-expressed genes for ranking the most likely gene expression regulators causal to eQTLs.

**Results:**

We have demonstrated that known co-expression and genetic interactions between C. elegans genes can be complementary in predicting gene expression regulators. Several algorithms were compared in respect to their predictive potential in different network connectivity contexts. We found that genes associated with eQTLs are highly clustered in a C. elegans co-expression sub-network, and their adjacent genetic interactions provide the optimal functional connectivity environment for application of the new SPF-based algorithm. It was successfully tested in the reverse-prediction analysis on groups of genes with known regulators and applied to co-expressed genes and experimentally observed expression quantitative trait loci (eQTLs).

**Conclusions:**

This analysis demonstrates differences in topology and connectivity of co-expression and genetic interactions sub-networks in WormNet. The modularity of less continuous genetic interaction network does not correspond to modularity of the dense network comprised by gene co-expression interactions. However the genetic interaction network can be used much more efficiently with the SPF method in prediction of potential regulators of gene expression. The developed method can be used for validation of functional significance of suggested eQTLs and a discovery of new regulatory modules.

## Background

Reconstruction of functional networks from the known pair-wise connectivity between biological molecules offers systems level insights into complex biological processes [1]. The topology of such a network is determined by all types of interactions used for its reconstruction and selected from: direct physical or regulatory protein interactions as well as indirect indicators of functional links between proteins. The indirect indicators such as genetic interactions, gene co-expression, co-occurrence, fusions are usually presented as probabilistic. Integrative analysis of different types of data is widely applied to construct regulatory gene networks [2, 3], overall improving the predictive power of such networks [4]. It has been shown that indirect indications of functional relevance between genes such as gene co-expression and genome co-localization are largely complementary and correlate well with ontology-based protein groupings [5, 6]. However, not all interactions correlate well: genetic and protein interactions barely overlap [7], that causes some obvious challenges in retrieving useful information from a reconstructed species-specific network. Network-based investigations require an accurate choice of data and significance thresholds to reflect a proper balance between the connectivity and the reliability of a network. Pitfalls are that not all types of connectivity data have been investigated to the same extent and the unequal availability of data for different organisms. Therefore a prior understanding of the impact of the different available data types on the topology of the generated networks is essential.

The gaps in experimentally-derived knowledge on regulatory and structural features of biological systems can be filled to some extent by theoretical predictions. For this study we propose a new application of a modified statistical algorithm [8], based on the “shortest path function” (SPF) to rank the regulators by their potential involvement with the genes in a co-expressed cluster. The suggested algorithm can also be applied to any explicitly defined group of genes.

One of the efficient methods that allow reconstruction of the regulatory interactions between genes is based on expression quantitative trait locus (eQTL) data derived from genetical genomics experiments. eQTL data has been used in several ways for network/pathway reconstruction [3, 9–11]. However these methods focused on a small number of genes or only used eQTL data without consideration of other available information on gene and protein connectivity. The advantage of using of eQTL related co-expression clusters is an opportunity to filter potential candidates by their genomic position. Here we present an algorithm which uses eQTL data in combination with published functional interactions in *C. elegans* [1]. By application to age-specific eQTL data for *C. elegans* [12] we show that it leads to reasonable predictions for the underlying regulatory genes. The suggested approach can refine interpretation of organism- specific integral biological networks and used for prediction of protein complexes and genetic regulators from a network context.

## Methods

### Data sets

#### Dataset for validation of gene clusters

For eQTL-hotspot gene selection we used previously published eQTL data [12], retrieved from WormQTL [13]. This experiment was done on three *C. elegance* worm age groups. In each of the 3 experimental age groups the genes with a shared regulatory locus were selected by taking all the genes having an eQTL with a *log*_10_(*p*)-value above 3 at the same locus (see Table 1).

**Table 1:** EQTL-hotspots associated with *C. elegance* age groups

EQTL-hotspot	Chromosome	Left marker	Right marker	Number of genes		
Juvenile worms						
1	I	4	6	261		
2	V	98	100	183		
Reproducing worms						
3	IV	61	63	131		
4	V	95	100	194		
Old worms						
5	II	37	40	144		
6	IV	61	65	164		
7	IV	68	68	92		
8	V	95	100	215		

WormBase WS220 [14] has been used for retrieval of gene names and IDs, associated functional annotations and ontological categories. WormNet [1] has been used to obtain pair-wise interactions between genes. WormNet contains connectivity data from *C. elegans*, *Drosophila melanogaster*, *Homo sapiens*, and *Saccharomyces cerevisiae*. Among the different types of data there are: co-citation, co-expression, protein physical interactions, protein complexes, genetic interactions. In our analysis we used complete Wormnet, which was denoted as ’wWormnet’ and Wormnet sub networks, comprised of the genetic interactions (’gWormnet’) or the gene co-expression connections in *C. elegans* (’eWormNet’).

#### Data set for testing predictive algorithms

To test our algorithms for detection of potential regulators from the gWormNet we used 3 groups of genes, each known to be regulated by 3 regulators highly ranked in our eQTL analysis (see Table 2). These groups of genes were retrieved from WormBase and complemented with their genetic interactions and co-expression data retrieved from WormNet.

**Table 2:** The gene groups with known regulatory genes

Group	Regulator	The number of genes	Genes
1	pop-1	14	egl-17, glr-1,
			end-3, end-1,
			sdz-23, ceh-22,
			sdz-26, wrm-1,
			psa-3, end-1,
			sod-3, end-3,
			sys-1, ceh-10
2	daf-2	8	daf-16, sgk-1,
			daf-21, fkb-3,
			dao-2, old-1,
			dao-3, dao-4
3	lin-11	9	odr-7, syg-1,
			cdh-3, ceh-2,
			syg-2, ast-1,
			egl-17, zmp-1,
			cog-1

#### Application of the SPF method to a new data set

To test our algorithm on a larger set of highly interconnected and co-expressed genes we selected a group of genes involved in translation that had a strong co-expression pattern in two *C.elegans* strains [15–17]. The micro-array data [18] were retrieved from NCBI’s Gene Expression Omnibus (GEO [19]) under GSE5395. By means of the Mev4 application [20] we performed clustering of the gene expression profiles by absolute mRNA values. By application of K-means clustering of the expression profiles we have produced a number of gene cluster arrays and have chosen the most robust cluster of genes (slightly changes depending on the requested cluster number) from a 50-cluster K-means analysis where it was composed by genes with highly homogenous expression values. This largest cluster (Cluster K1) enriched for highly co-expressed genes relevant to translation was selected for further analysis. String software [21, 22] has been used for visualization of graphical networks reconstructed for sets of *C. elegans* genes.

### Methods

#### Statistical validation of the gene clusters

To investigate WormNet connectivity properties of the selected gene clusters we have used quite a standard approach based on calculation of gene pairs (*GP*) connected in a cluster (or a module) [2, 4]. But in contrast to common methods, where the fraction of direct links among all links is defined, we calculate this fraction as dependent on a random set of genes (nodes) with the same degree distribution in a network (further, we use the notation referred to as “random cluster” for such a set). The respective values of statistical significance (GP score) can be defined as

(1) $$\begin{array}{@{}rcl@{}} GP score=\frac{GP-<GP>_{random}}{\sigma(<GP>_{random})} \end{array} $$

Where <*GP*>_*random*_ and *σ*(<*GP*>_*random*_) are the mean and the deviation of *GP* in ensemble of random clusters defined in a given network. Note that the use a set of nodes of the same degree distribution is a quite natural “null model”, arising in the study of motif‘s distribution [23] or of the specificity and the stability in a topology of networks [24]. Another approach to characterize the connectivity of a gene cluster is based on the so-called the shortest path function (SPF): where the shortest path along the network from a given vertex node *i* to some another vertex node *j* is denoted by *d*_*i*,*j*_. The SPF for a given cluster is determined by the following

(2) $$\begin{array}{@{}rcl@{}} k^{SPF}_{cl}=\sum\limits_{i,j=1}^{N}\frac{1}{d_{i,j}} \end{array} $$

**Fig. 1:**
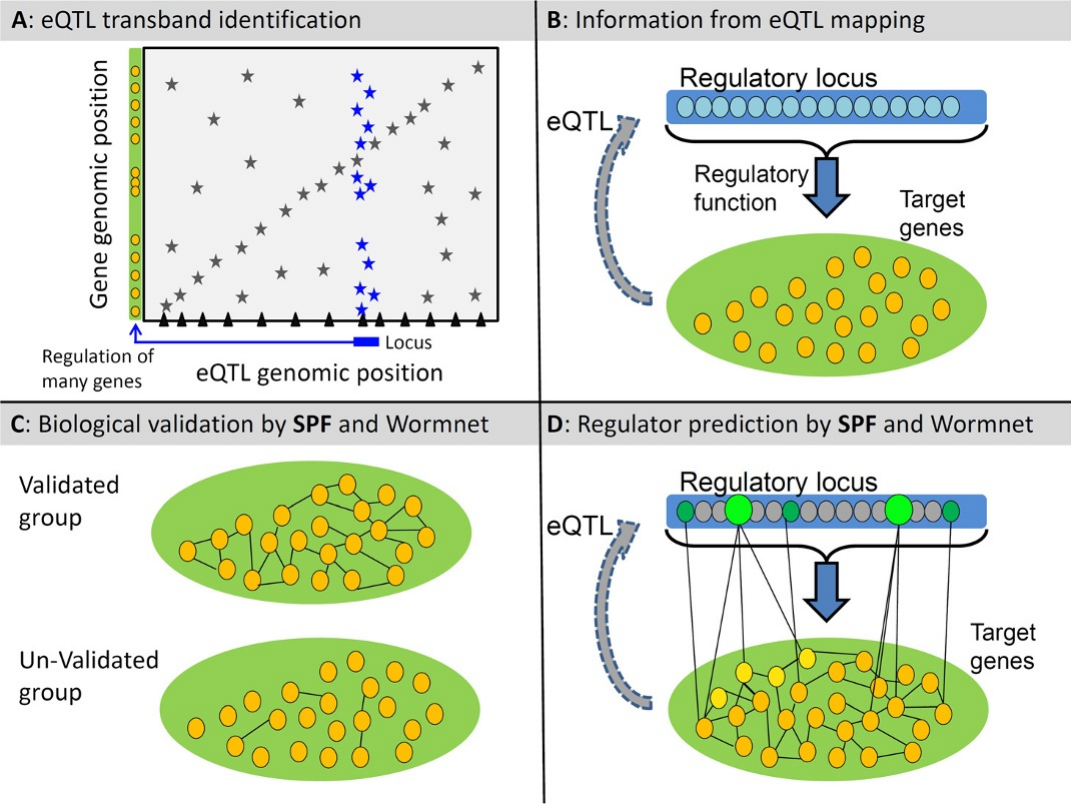
Candidate regulator prediction for natural variation in gene expression regulation using the SPF method. Genetical genomics (eQTL mapping) identifies loci involved in the regulation of gene expression. In these experiments eQTL-hotspots (trans-bands) can be identified, which indicate loci regulating the expression of many genes. **a** An outcome of a genetical genomics experiment is represented schematically. The genes for which the expression levels where measured in a recombinant inbred line population are shown on the Y-axis. The x-axis shows the location of the eQTL peak position (potential regulatory loci). The blue locus is an example of an eQTL-hotspot, corresponding to a position of a putative regulator of multiple genes (shown in blue). **b** By eQTL mapping we have obtained two types of information which can be used to identfy the regulatory gene. Firstly target genes are identified as having an eQTL at a particular genomic location. Secondly, the locus harbouring many eQTL is likely to contain a gene affecting expression of multiple targets. Therefore it is very likely that the candidate gene has a regulatory function, for example, a transcription factor or a receptor. **c** In many cases eQTL hotspot loci contain > 100 genes [12] and validation is important before pursuing the potential regulator. The SPF method can be used to validate eQTL hotspot by investigating if the genes mapping to the eQTL hotspot share a relationship based on hundreds of experiments categorised in WormNet [34]. A validated group of genes will have more connections in WormNet compared to a random group. Thereby the SPF method can identify false-positive eQTL hotspots, for example caused by experimental variation. D: The identification of potential regulators is laborious [29], and candidates prioritizing is imperative. A validated group of co-regulated genes can be used to predict the most likely regulator by selecting genes on the eQTL hotspot locus with the most direct connections to the target genes (*dark orange circles*), or indirect connections via other genes (*yellow circles*)

Thus defined, the SPF has a very transparent meaning, since it defines the sum of lengths of reciprocal paths between a pair of genes. If *i* and *j* are not linked within the network, the contribution to the SPF from this pair (*i*,*j*) equals zero. Whereas, for a directly linked node pair the contribution reaches its maximum (2). So, the SPF can be used to quantitatively characterize the connectivity of a gene cluster within a given network (see Fig. 1).

As for the gene pairs, we compare the SPF coefficients calculated for gene clusters and for random sets of nodes with the same degree distribution in a network. Apparently, direct links contribute to the calculated SPF coefficient the most; however for quite rare networks a contribution of longer paths can be significant.

#### Prediction of potential regulators

The methods for prediction of potential gene expression regulators from a network are usually based on the enrichment of direct links [2] or the overlap of the sub network of the genes directly connected to the potential regulator with the studied gene cluster [4]. Our approach to predict the potential gene expression regulators from a network context is based on the assumption that potential regulators of a cluster are associated with the cluster by multiple genetic connections. The SPF coefficients $k^{SPF}_{M}$, determined as

(3) $$\begin{array}{@{}rcl@{}} k^{SPF}_{M}=\frac{1}{N}\sum\limits_{i=1}^{N}\frac{1}{d_{i,M}}  \end{array} $$

is proved to be promising identifier of potential regulators of co-expressed gene clusters. Note that a potential regulator that belongs to the cluster or lie outside of it, can have connections to *not all* cluster genes. It should however have more connections to the cluster than to other genes in the network, where the connections can be non-direct. As before, we analyze the correlation of the SPF coefficients and a respective number of direct links between a potential regulator and the cluster genes.

## Results

### Statistical properties of co-expression clusters

#### Statistical validation of eQTL hot spot gene clusters

Our first goal was to define the topological properties of the eQTL hot spots in a network. We calculated the SPF coefficients for the trans-regulatory hotspots (or trans-bands) associated with ageing in *C.elegans* (see Table 1) in eWormNet (Fig. 1). Table 3 demonstrates the values of GP scores defined by (1) and the respective GP scores in the SPF coefficients. The SPF coefficients for *trans*-bands (numbering according to Table 1) are slightly smaller than the respective GP scores. This indicates a nearly homogeneous distribution of the shortest paths in eWormNet. However, the number of direct links (*d*_*i*,*j*_=1) is contrasting between the eQTL hotspots and random clusters for all *trans*-bands (the respective scores are more than 1). Therefore, we can conclude that the eQTL *trans*-bands are characterized by higher connectivity at the level of direct links in eWormNet.

**Table 3:** Statistical significance scores (1) for number of direct links (gene pairs GP) and the SPF coefficients for eQTL hotspot gene clusters

Cluster	GP score	the SPF score
1	114.8	100.2
2	15.8	6.2
3	22.2	10.8
4	25.8	9.3
5	185.2	96.4
6	5.5	5.34
7	1.6	35.70
8	4.3	0.88
K1	384.7	248.8

The same analysis was applied to the co-expression cluster of genes involved in translation (Cluster K1). We found that the genes within the cluster have a higher connectivity both in SPF and in the number of direct links Table 3. Again, we can confidently distinguish the co-expression gene cluster by only on the number of the comprised direct links, with the value of the respective statistical significance is order of 100.

Although we cannot distinguish both the cluster of co-expressed genes and eQTL hotspots from a random gene set by the values of the SPF coefficient alone, this method can be successfully applied to predict potential regulatory genes. We assume that a cluster‘s regulatory gene must be well-connected with all genes in the cluster, rather than directly linked to a small subcluster. Therefore the calculation of a SPF coefficient (averaging the lengths of all paths to a cluster) should be helpful and interpretive.

### Prediction of expression cluster regulators

#### Testing of the SPF method

We have used the 3 pre-selected gene groups with known regulators (Table 2) to test our algorithm on prediction of potential regulators. Two different methods have been tested. We consider every gene in a network as a potential regulator and rank them by the fraction of direct links (FDL), which a potential regulator has to genes in a co-expression cluster. To be efficient, this method requires a dense connectivity matrix. The predicted regulators were expected to have a strong and specific involvement in modulation of expression of at least part of the gene cluster. A gene with any function could have an effect on other gene expression. However, we expect to define a direct regulator of the mRNA pool from the set of highly ranked genes. We used a number of ontological categories, such as transcription, splicing, mRNA degradation and transport functions to filter the best candidates.

The second method was based on ranking of the network nodes by their average distance to all cluster genes, which was defined by the SPF. The different connectivity subnetworks reconstructed for genes in a cluster had different topologies, with the genetic interactions subnetwork (gWormNet) generally being the least connected. The SPF method did not require a matrix to be dense and could be applied to a gWormNet. The power of the SPF method was in its compensation for fragmentary connectivity data, as the top ranked regulatory links would be projected on the rest of the gene cluster.

Both the FDL and SPF methods were applied to the wWormNet and gWormNet subnetworks reconstructed for the 3 test clusters (Table 4). We ranked all genes in WormNet as potential regulators to a given gene group. Well studied regulators had high ranks in both wWormNet and gWormNet subnetworks with *pop-1* and *daf-2* showing the largest number of direct links to their gene groups (Table 4). Their coefficients in eWormNet were equal to zero (not shown in Table 4), indicating that the identified regulators would be unlikely co-expressed with the regulated groups of genes. Application of the SPF algorithm increased the rank of the regulator *lin-11* from the 19^*th*^ to 8^*th*^ position for the test cluster 3 in wWormNet, and from 3^*rd*^ to 1^*st*^ position in gWormNet, proving that the method may be successfully applied to even a small group of *C. elegans* genes connected in WormNet.

**Table 4:** The ranks of potential regulators of the gene groups in Table 2

Group	Regulator	FDL	SPF	FDL	SPF	
		wWormNet	wWormNet	gWormNet	gWormNet	
1	pop-1	1	1	1	1	
2	daf-2	1	1	1	2	
3	lin-11	19	8	3	1	

Comparison of computational extrapolations made via applications of the wSPF and gSPF methods demonstrate robustness of the highest and also other highly ranked predictions. Two other suggested regulators for the *pop-1* regulated test gene group (*mom-2* and *skn-1*), were ranked second and third by both methods, and 6 regulators suggested for *daf-2* test gene group were also among the top 10 predicted by application of the wSPF or gSPF. Three other regulators for emphlin-11 test group (*lin-1*, *lin-29* and *egl-38*), were also highly ranked by both the wSPF and gSPF scores.

To address problems with potential false-positive outcomes in the application of SPF-based algorithms, we have compared the scores and inter-connectivity of the top regulators predicted for each test gene group. This comparison showed that the very top ranked regulators occurred to have strongly distinguished increments in their ranking scores compared to the other suggested regulators, making them to stand out. For instance, the gSPF score of *daf-2’s* is 0.94, which is high compared to the second ranked (*daf-7’s*- 0.69), the third ranked (*age-1’s* -0.67) and the eighth ranked (*daf-16’s* -0.56). The score of *daf-2’s* in wSPF equals 1 and for both *daf-7* and *age-1*, now ranked as the 8^*th*^ and the 9^*th*^, scores equal at 0.69. The gSPF scores for the three top ranked regulators of the *pop-1* associated gene group are 0.55, 0.50, and 0.50 respectively, whereas the score of the fourth ranked gene decreases abruptly to 0.37. Their corresponding scores using wSPF are: 0.78, 0.65, and 0.63. *Lin-11* shares the gSPF score of 0.5 with *lin-29*, however *lin-11* has a higher score (0.73 versus 0.67) using wSPF.

The top suggested regulators that have high scores in both gSPF and wSPF ranking are also strongly inter-connected in a network. *age-1*, *daf-7*, and *Pdk-1* that followed *daf-2* in the gSPF prediction list are the most connected to *daf-2* by the number of experimentally supported links. On the other hand, *dao-5*, *dao-6* and *isp-1* were only higly ranked using the wSPF method and have lower positions in the gSPF list. These genes did not have experimentally defined connections to *daf-2* or its immediate connectors. Functions of *dao-5*and *dao-6* are also linked to regulation of dauer stage of larva development, which may explain their high over-all connection to the dauer-associated *daf-2*-regulated test gene group on wSPF. The gene *isp-1* which is a component of the respiratory chain and probably is associated with the test group of genes via co-expression connections taken in consideration only in wSPF analysis. In case of the *lin-11*-regulated gene group all the other highly ranked regulators contributed to cell differentiation and egg laying and can be potentially functionally-relevant. wSPF however could generate potentially false predictions, such as of the gene B0034.1 ranked as 3^*rd*^ by wSPF and having ‘0’ ranking score in gSPF.

From this analysis we conclude that there is no critical false-positive issue with respect to reliability of SPF method-based predictions, especially if the utilized network context is comprised of only experimentally validated genetic interactions. Parallel application of wSPF and gSPF can help to refine the predictions by contrasting true functional regulators among at least top 10 ranked genes.

#### Prediction of regulators for eQTL hotspots

Subsequently we applied the SPF method to the age-associated *C. elegance* eQTL data (Table 1) [13, 16]. Application of the SPF method to a gWormNet led to promising regulatory predictions for eQTL-hotspots. Four regulators could be predicted for the eQTL-hotspot on the left arm of chromosome I in the juvenile (L4) group when selection included the position of the eQTL-hotspot locus. Interestingly, 3 from top 4 suggested regulators (*Pop-1*, *xnp-1*, *lin-17* and *lin-44*) are related to WNT pathway (see Table 5). *Pop-1* also associates with the chromosome V eQTL-hotspot in juvenile but cannot be the first-order causal regultor of this QTL-hotspot as it is not located on the chromosome V locus. When the location of the regulators was not considered, we found that both *daf-2* and daf-16 were associated with the two juvenile eQTL-hotspots possibly functionally linking these loci to wnt signaling. *Age-1* was suggested for the eQTL-hotspot on chromosome II, specifically found in old worms, by the analysis of the gWormNet (see Table 5). No regulator could be identified for the eQTL-hotspot on the far right arm of chromosome V, found in all three age groups, even though the genes in this eQTL-hotspot were highly linked in wWormNet. This could mean that a relatively less well studied gene is involved in this eQTL regulation.

**Table 5:** Top regulators for eQTL-hotspot gene groups predicted by the SPF method in gWormNet

EQTL-hotspot	Chromosome	Gene	Function			
Juvenile worms						
1	I	*pop-1*	TCF/LEF TF, WNT pathway			
1	I	*xnp-1*	DNA helicase, stress response			
1	I	*lin-17*	Wnt signaling			
1	I	*lin-44*	Wnt signaling			
Old worms						
5	II	*age-1*	PI3K, daf-2 Insulin pathway			

Application of the SPF method to wWormNet gave more diverse results presented in Table 6. Besides a long list of candidate genes with unknown functions there were promising predictions of steroid-hormone receptors *nhr-218* and *nhr-269*, linked to let-60 and thus to ras and wnt signaling for the Chromosome V eQTL-hotspot in old worms, and also a prediction of RNA binding protein modulator encoded by *moe-3* for the chromosome II eQTL-hotspot in old worms.

**Table 6:** Top regulators for eQTL-hotspot gene groups predicted by the SPF method in wWormNet

EQTL	Chrom.	Gene	Function			
Juvenile worms						
1	I	K09H9.2,	Endocytosis/			
		*clec-53*	regulation of growth rate			
		R12E2.2				
1	I	W01B11.1				
1	I	*sep-1*	Cell division			
1	I	*mis-12*	Cell division			
1	I	Y54E10BR.3	TF/Zn ion binding			
1	I	Y71F9B.6				
2	V	*fbxa-192*	Protein interaction			
2	V	*str-92*				
2	V	T10C6.7	Protein interaction			
2	V	Y59A8A.3				
Reproducing worms						
3	IV	Y55F3BL.2	Metal ion transport			
3	IV	Y69A2AR.16	Metabolism/oxidoredutase			
3	IV	Y69A2AR.21	Embrionic development			
4	V	Y32B12A.5				
4	V	Y43F8B.13				
4	V	Y43F8B.14				
4	V	Y51A2B.4	Lipid metabolism			
4	V	Y70C5B.1				
4	V	*srh-296*	Integral membrane component			
Old worms						
5	II	*moe-3*	RNA binding/iRNA modification			
5	II	Y17G7B.18	Positive regulation of growth rate/development			
5	II	*cpt-1*	Acetyl-transferase/histone modification			
5	II	*csp-1*	Caspase/apoptosis			
5	II	*pqn-87*	Prion/protein modification			
6	IV	F15E6.4				
6	IV	F28F9.3				
6	IV	T08B6.4				
6	IV	Y9C9A.1	Structural element of vitelline membrane			
7	IV	C17H12.12	Protein binding			
7	IV	C17H12.5	Tyrosine phosphatase			
7	IV	C31H1.1				
7	IV	F36H12.5				
7	IV	F38A5.6				
7	IV	ZK354.3				
8	V	Y38H6C.15				
8	V	Y38H6C.18				
8	V	*tgt-2*	Queuine tRNA-ribosyltransferase activity modification			
8	V	T26E4.10	Lipid storage			
8	V	T26F2.2				
8	V	*sri-7*	Integral membrane component			
8	V	*nhr-218*	TF,steroid hormon receptor			
8	V	*str-151*	Integral membrane component			
8	V	*nhr-269*	TF,steroid hormon receptor			

#### Predictions for the co-expression cluster K1

Both algorithms were applied to the cluster of co-expressed genes involved in translation (Cluster K1). Among the most promising predicted regulators are: *daf-2*, *iff-1*, *cgh-1*, *tin-9.2* and *car-1*. They all are related to mRNA processing/translation/decay and are in a cross-talking relationship (see Table 7). The SPF method allowed us to predict some regulators which we could not detect by the FDL method. Especially, it was demonstrated on gWormNet, where the density of network links is low (see Table 8). We could identify a number of genes encoding TFs that may be considered for a role of transcription regulators of genes in Cluster K1, such as: *taf-5*— transcription initiation factor TFIID subunit 5, *xbp-1*— heat-shock transcription factor, *sin-3* — histone deacetylase subunit and premRNA-splicing factor *cwc-22*. Compared to FDL, SPF greatly increased the ranking position of *daf-2*, genes upstream *daf-2* (C25A1.10) or ones that were known to be directly affected by *daf-2* mutation (C05C8.3).

**Table 7:** Top regulators for test cluster K1 predicted by the FDL and the SPF methods in wWormNet

Seq. IDs	Gene	Function		
F57B9.6	*inf-1*	Transl.initiation/ RNA transport		
T05G5.10	*iff-1*	Transl.initiation/ NMD		
Y71G12B.8	Y71G12B.8	RNA helicase/ RNA transport		
T10C6.14, T10C6.12, T10C6.11,	38 His genes	Histones		
F45F2.4, F45F2.12, ZK131.4,				
ZK131.6, ZK131.8, ZK131.10,				
K06C4.10, K06C4.11, K06C4.4,				
K06C4.3, K06C4.12, ZK131.1,				
K06C4.2, F35H10.1, F17E9.12,				
F17E9.13, C50F4.7, K03A1.6,				
C50F4.5, F08G2.2, B0035.9,				
B0035.7, F07B7.9, F07B7.10,				
F07B7.4, F07B7.3, F07B7.11,				
F54E12.3, F54E12.5, F55G1.11,				
F55G1.10, F22B3.1,H02I12.7,				
T23D8.5, T23D8.6, F45F2.3				
C41D11.2	*eif-3.H*	Transl.initiation		
F32E10.1	*nol-10*	Nucleolar protein, polyglut. binding		
F54H12.6	*eef-1B.1*	Elongation factor		
C01F6.5	*aly-1*	RNA export		
M163.3	*his-24*	Histones		
B0564.1	*tin-9.2*	Decay/ NMD		
Y18D10A.17	*car-1*	Decay/decapping		
F56D12.5	*vig-1*	RISC component/miRNA binding		
F26D10.3	*hsp-1*	Splicing		
R04A9.4	*ife-2*	Transl.initiation

**Table 8:** Top regulators for the test cluster K1 predicted by the SPF method in gWormNet

Seq. IDs	Gene	Function		
Y55D5A.5,B0334.8,Y116F11B.1	*daf-2*, *age1*, *daf-28*	Insulin/aging		
F35H8.5	*exc-7*	mRNA processing		
W10D5.1	*mef-2*	TF		
C17D12.2	*unc-75*	Splicing		
C47G2.2	*unc-130*	TF		
F30F8.8	*taf-5*	Transl.initiation		
R74.3	*xbp-1*	TF, histone modulation		
F33A8.1	*cwc22*	Splicing		
C41C4.4	*xre-1*	(RNA processing) decay/processing		
C37H5.8	*hsp-6*	Decay		
C26D10.2	*hel-1*	DNA helicase		
C07H6.5	*cgh-1*	Decay/ decapping		
F02E9.4	*sin-3*	Histone modulation		
M163.3	*his-1*	Histone		
212312 C25A1.10	*dao-5*	rRNA transcription/aging		
ZC247.3	*lin-11*	TF		
R107.8	*lin-12*	TF		
C05D9.5	*ife-4*	Transl.initiation		
R11E3.6	*eor-1*	TF		
F43G9.11	*ces-1*	TF		
ZK909.4	*ces-2*	TF		

**Fig. 2:**
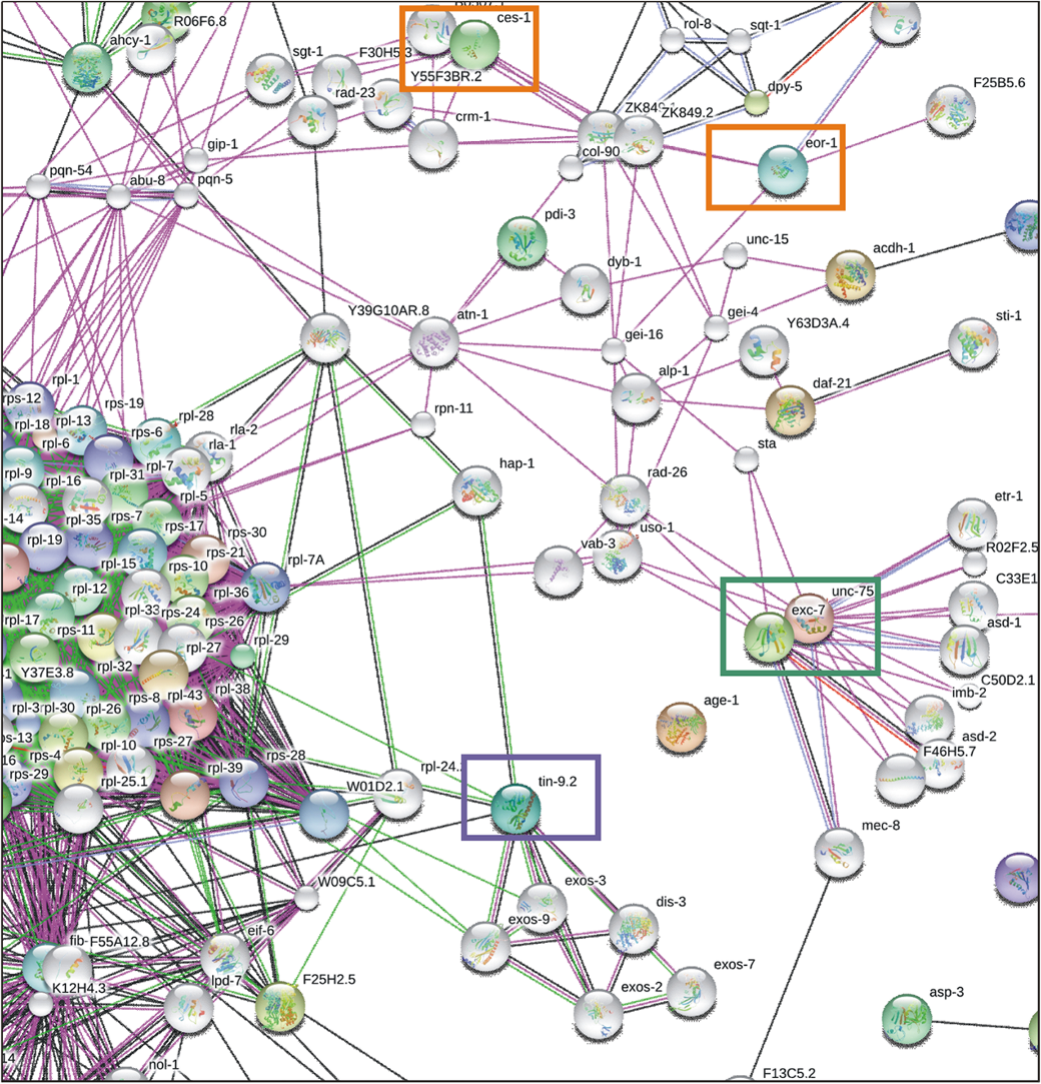
Connectivity between the predicted regulators and the cluster K1 in STRING Network browser: experimentally derived interactions (*pink*), co-expression (*black*), co-localization in the genomes (*green*), and co-occurrences in the genomes (*blue*). Colored circles represents input genes, white circles — the most associated additional nodes (set number of 200) automatically added by a STRING software on a request to increase a connectivity between uploaded functions. Predicted potential regulators are shown in frames: orange — the SPF method, purple — the FDL method, green node excluded in hub-exclusion SPF method

Figure 2 illustrates typical positions of the predicted regulators for the Cluster K1. Nodes predicted by the FDL method (purple frame) are located proximal to the cluster or even inside the cluster. The nodes predicted by the SPF method can be distant from the many nodes in the cluster (*ces-1*, *eor-1*, orange frames in Fig. 2). Though the connections between the SPF-predicted node and the cluster may include several intermediate steps, the majority of these steps do contain the nodes that can translate signals at the level of mRNA pool regulation, potentially representing complexes of proteins with a joint regulatory performance.

## Discussion

Our study aimed to refine algorithms that use biological networks for identification of gene regulators. We used test clusters of co-regulated genes with known regulators and a large cluster of co-expressed house-keeping genes to validate their performances. Both test computations gave us positive outcomes for an application of the SPF-function based algorithm, especially when only genetic interactions were used for network reconstructions. We were able to perform a reverse prediction of the regulators for the selected clusters of co-regulated genes and to suggest a number of expected functional links/potential regulators of the cluster of co-expressed genes relevant to translation.

Our method may be especially useful in finding the causal regulators for gene expression QTLs in genetical genomics studies. Genes sharing an eQTL are very likely to have a common regulator as well as a joined biological function, however, candidate regulators in the relevant genomic position are still too numerous for a focused experimental validation. As more diverse organisms like yeast [4, 25, 26], plants [3, 9, 10, 27], animals [12, 18, 28–32], human [33] are interrogated via eQTL analysis, an efficient way of candidate gene selection is indeed becoming essential. This application is also of potential interest for interpreting the results of population genomic studies, since eQTLs from individual experiments may provide inconclusive clues to the relevant functional relationships underlying observed responses. Our methods predict the most likely regulator based on hundreds of previously published experiments, as, in our case, are those used to generate WormNet [34].

**Fig. 3:**
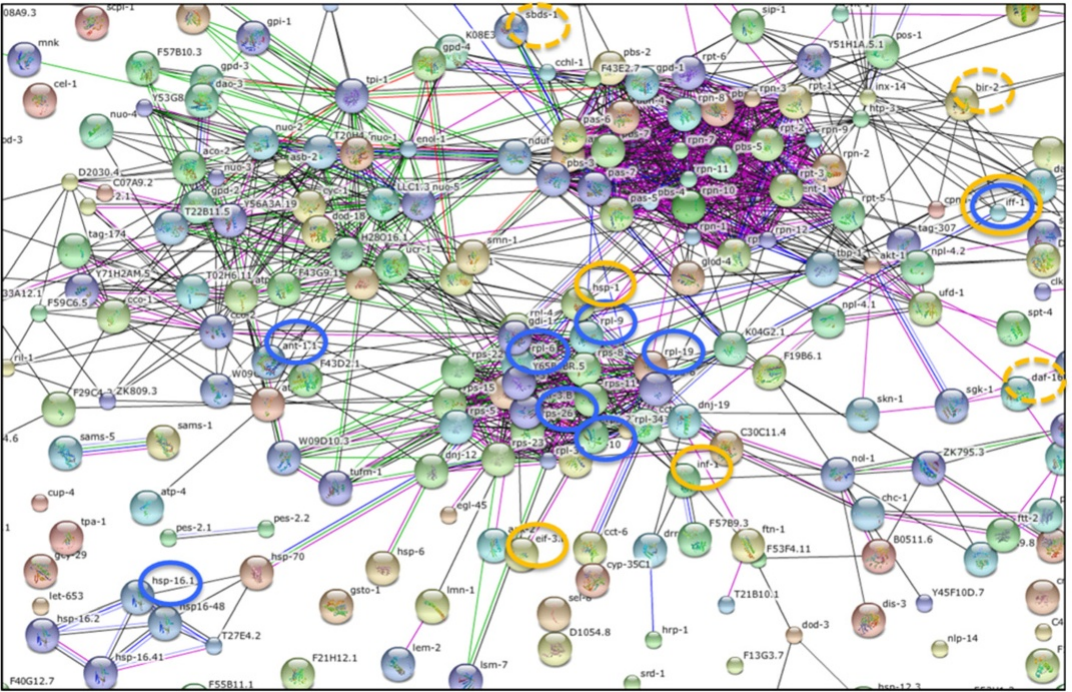
Network reconstructed from the *C.elegans* genes with an adult life span phenotype from WormBase 220. Three main distinguished clusters can be seen: in the center — ribosomal, top left —metabolic, top right — proteosome and exosome functions. Blue circles indicate the test Cluster K1 genes. Orange-predicted regulators, dashed borders — functionally associated regulators discussed in the manuscript. (Not all aging-related functions related to the Cluster K1 are shown on this figure)

The test co-expression gene Cluster K1 mainly contained genes involved in the translational machinery. Our analysis highlighted its primary association with insulin-dependent pathway via such suggested regulators as *daf-2* and insulin-regulated mRNA decay functions *iff-1* and *bir-2* [35]. The insulin pathway has an established role in the regulation of translation [36, 37]. As it is involved in regulation of the aging process, and *iff-1* was shown to have a longevity phenotype, we investigated the genes of cluster K1 for association with longevity phenotypes (Fig. 3). The analysis has produced a supportive outcome. Predicted K1 connections, *iff-1* and *tin-9.2*, are associated in a network with a ribosome maturation protein SBDS [38, 39], which is required for the longevity phenotype of *daf-2* [40]. Interestingly, the transcription factors predicted for Cluster K1 by the SPF method were also found to be involved in regulation of longevity. The genes *cgh-1* [41], *dao-5* [42], *hel-1* [43] were already linked to aging processes downstream of *daf-2*, *daf-16*, and in case of *dao-5*, to a *daf-16* independent pathway associated with determination of the adult life span GO-term in WormBase database.

All top regulators predicted for age-associated eQTLs were relevant to aging and longevity processes. Finding AGE-1 as a possible regulator for an eQTL-hotspot expressed in old age worms is especially interesting as this protein gene has been already suggested as a regulator of lifespan after heat shock [44]. POP-1, a predicted regulator of the chromosome I eQTL-hotspot in juveniles, is a TF that functions as a component of WNT signaling pathways [36], and both longevity-related DAF-2 and DAF-16 are known to interact with its components [45]. For instance, it was shown that DAF-2, DAF-16 and POP-1 synergistically affect the immune response in *C. elegans* [46].

We anticipate that our work provides new insights to the structure of biological functional networks and highlights the aspects that need to be considered in the prediction of regulatory nodes and functional modules from a multilevel and heterogeneous network context.

## Conclusions

1. Application of the SPF function has been adopted for computational prediction of potential gene expression regulators from a network context.
2. Computational identification of groups of co-expressed genes in a network was proven to be achievable. The developed method can be used for validation of functional significance of suggested eQTLs and a discovery of new regulatory interactions.
3. We have demonstrated differences in topology and connectivity of co-expression and genetic interactions subnetworks in WormNet. The modularity of less continuous genetic interaction network does not correspond to modularity of the dense network comprised by gene co-expression interactions. However the genetic interaction network can be used much more efficiently with the SPF method in prediction of potential regulators of gene expression.
4. Regulators predicted for the test cluster of co-expressed genes related to translation revealed the relation of this gene group to longevity. RNA decay may be suggested as an important player in longevity regulation.
